# Chemopreventive Effects of Dietary Isothiocyanates in Animal Models of Gastric Cancer and Synergistic Anticancer Effects With Cisplatin in Human Gastric Cancer Cells

**DOI:** 10.3389/fphar.2021.613458

**Published:** 2021-04-08

**Authors:** Hanne-Line Rabben, Yosuke Kodama, Masahiko Nakamura, Atle Magnar Bones, Timothy Cragin Wang, Duan Chen, Chun-Mei Zhao, Anders Øverby

**Affiliations:** ^1^ Department of Clinical and Molecular Medicine, Norwegian University of Science and Technology (NTNU), Trondheim, Norway; ^2^ The Central Norway Regional Health Authority, Stjørdal, Norway; ^3^ Center for Clinical Pharmacy and Clinical Sciences, School of Pharmacy, Kitasato University, Tokyo, Japan; ^4^ Cell, Molecular Biology and Genomics Group, Department of Biology, Norwegian University of Science and Technology (NTNU), Trondheim, Norway; ^5^ Division of Digestive and Liver Diseases, Columbia University College of Physicians and Surgeons, New York, NY, United States

**Keywords:** dietary (or plant) isothiocyanates, gastric cancer, glutathione, glutamine, cisplatin, mice

## Abstract

Naturally occurring isothiocyanates (ITCs) from edible vegetables have shown potential as chemopreventive agents against several types of cancer. The aims of the present study were to study the potential of ITCs in chemoprevention and in potentiating the efficacy of cytotoxic drugs in gastric cancer treatment. The chemoprevention was studied in chemically induced mouse model of gastric cancer, namely N-methyl-N-nitrosourea (MNU) in drinking water, and in a genetically engineered mouse model of gastric cancer (the so-called INS-GAS mice). The pharmacological effects of ITCs with or without cisplatin were studied in human gastric cell lines MKN45, AGS, MKN74 and KATO-III, which were derived from either intestinal or diffused types of gastric carcinoma. The results showed that dietary phenethyl isothiocyanate (PEITC) reduced the tumor size when PEITC was given simultaneously with MNU, but neither when administrated after MNU nor in INS-GAS mice. Treatments of gastric cancer cells with ITCs resulted in a time- and concentration-dependent inhibition on cell proliferation. Pretreatment of gastric cancer cells with ITCs enhanced the inhibitory effects of cisplatin (but not 5-fluorouracil) in time- and concentration-dependent manners. Treatments of gastric cancer cells with PEITC plus cisplatin simultaneously at different concentrations of either PEITC or cisplatin exhibited neither additive nor synergetic inhibitory effect. Furthermore, PEITC depleted glutathione and induced G_2_/M cell cycle arrest in gastric cancer cells. In conclusion, the results of the present study showed that PEITC displayed anti-cancer effects, particularly when given before the tumor initiation, suggesting a chemopreventive effect in gastric cancer, and that pretreatment of PEITC potentiated the anti-cancer effects of cisplatin, possibly by reducing the intracellular pool of glutathione, suggesting a possible combination strategy of chemotherapy with pretreatment with PEITC.

## Introduction

Gastric cancer is one of the leading causes of cancer in the world with over one million new cases reported in 2018 (GLOBOCAN) (Ferlay et al., 2013; Bray et al., 2018). Despite dramatic decline in gastric cancer incidences in later years, gastric cancer is the fifth most common cancer with a 5-year survival rate below 25%, making gastric cancer the third leading cause of cancer mortality worldwide (Ferlay et al., 2010; Ferlay et al., 2013). Chemoprevention of gastric cancer is to chemically prevent or delay the occurrence of malignancy. Although *Helicobacter pylori (H. pylori)* eradication can be an effective preventive method due to the putative pathogenic mechanisms, the chemoprevention using natural, synthetic or biological agents has enormous potential, given the high incidence together with the healthcare costs of treatment (Steward and Brown, 2013; Tan and Wong, 2013; Dunn et al., 2016). The treatments of gastric cancer include surgery, and chemotherapy regimens with either mono-chemotherapy (using single drug) or combination-chemotherapy (e.g., fluoropyrimidines and platinum-based therapies) for inoperable or metastatic gastric cancer (Van Cutsem et al., 2006; Cunningham et al., 2008; Koizumi et al., 2008; Kang et al., 2009; Orditura et al., 2014). However, patients with unresectable advanced gastric cancer usually have poor outcomes with median survivals of 10–18 months. Nearly half of patients with resectable gastric cancer have a recurrence and median survival is about 6 months (Leiting and Grotz, 2019). Thus, a challenge for improving patient care of gastric cancer in terms of survival and quality of life appears to be ineffective cytotoxic chemotherapy. These facts indicate that there are still great needs for improvement in the prevention and treatment of gastric cancer. Previously, we have showed that denervation (surgically, pharmacologically or genetically) suppressed the tumorigenesis of gastric cancer, which was associated with a decrease in WNT/β-catenin signaling, the suppression of stem cell expansion through M3 receptor-mediated cholinergic signaling and the reversion of metabolic reprogramming, and that the combination of denervation and mono-chemotherapy led to an enhanced effect on tumor growth and survival in an animal model of gastric cancer (Zhao et al., 2014; Rabben et al., 2016). Recently, we have further shown that neural signaling modulated metabolism of gastric cancer, reflected by metabolic switch from glutaminolysis to OXPHOS/glycolysis and normalization of the energy metabolism after denervation (Rabben et al., 2021). In the present study, we wanted to explore the potential of a class of anti-cancer agents, isothiocyanates (ITCs) for chemoprevention and enhancement of chemotherapy as they are also shown to interfere with tumor metabolism (Conaway et al., 2002; Lv et al., 2020).

Naturally occurring isothiocyanates (ITCs) are electrophilic plant phytochemicals derived from glucosinolates of edible vegetables such as broccoli, cauliflower, brussels sprouts, and cabbage. Phenethyl isothiocyanate (PEITC) has been tested in *in vitro*, *in vivo* and in clinical trials to study about the potential effects of prevention and treatment of different types of cancer (Hu and Morris, 2004; Keum et al., 2004; Keum et al., 2005; Clarke et al., 2008; Chu et al., 2009; Wu et al., 2009; Li et al., 2010; Tomczyk and Olejnik, 2010; Zhang, 2010; Chung et al., 2013; Li and Zhang, 2013). The aims of the present study were to study whether ITCs could prevent gastric tumorigenesis and whether ITCs could enhance the inhibitory effect of mono-chemotherapy on gastric cancer; and if so, to investigate the possible underlying mechanisms. To these ends, we utilized chemically induced mouse model of gastric cancer, i.e., N-methyl-N-nitrosourea (MNU) in drinking water, and genetically engineered mouse model of gastric cancer (the so-called INS-GAS mice) for studying chemoprevention, and used human gastric cancer cell lines, i.e., MKN45, AGS, MKN74 and KATO-III derived from either intestinal or diffused types of gastric carcinoma for studying the pharmacological effects of ITCs with or without cisplatin *in vitro*.

The possible mechanisms underlying the anti-cancer effects of ITCs have been suggested to involve inhibition of cytochrome P450 enzymes, induction of phase II detoxification enzymes, such as glutathione S-transferase (GST) and apoptosis, and cell cycle arrest, inhibition of migration, disruption of microtubules, and dysregulation of signaling pathways including major regulators such as WNT/β-catenin signaling pathway, NRF2, ERK, Jun and Akt signaling pathways (Yang et al., 2010; Gupta et al., 2014; Øverby et al., 2014; Lawson et al., 2015; Chen et al., 2018). In addition, glutathione (GSH) is a powerful regulatory tripeptide with antioxidant function that protects cells from oxidative stress by removing free radicals and peroxides. We and others have shown that ITCs conjugate with GSH, leading to depletion of GSH, elevation of oxidative stress and expression of GST-encoding genes, and that there are close relationship between glutathione and the levels of glutamine and glutamate in the cell-pool important for redox homeostasis (Øverby et al., 2015). Thus, we hypothesized that ITCs would enhance the cytotoxicity of cisplatin by depleting cells of GSH, and thus measured the levels of GSH and the ratio between glutamine and glutamate in connection with cell growth inhibition after treatment of ITCs.

## Materials and Methods

### Animals and Experimental Groups

All mice at ages between 1–12 months were housed three to four mice per cage on wood chip bedding with a 12 h light/dark cycle, room temperature of 22°C and 40–60% relative humidity at the standard housing conditions in a specific pathogen-free environment. Ninety mice including 54 wild-type (FVB) mice and 26 INS-GAS mice were divided into the following experimental groups: FVB mice (*n* = 16, eight male, eight female), FVB mice + MNU (*n* = 11, five female, six male), FVB mice + MNU + prePEITC (*n* = 16, eight female, eight male), FVB mice + MNU + postPEITC (*n* = 11, five female, six male), INS-GAS mice (*n* = 24, 10 female, 14 male), and INS-GAS mice + PEITC (*n* = 12, six female, six male). In each experiment, mice were randomly divided into different subgroups with gender-balance.

### Treatment of Phenethyl Isothiocyanate in a Chemically Induced Mouse Model of Gastric Cancer

The chemically induced gastric cancer model (FVB + MNU) was established according to our previous report (Zhao et al., 2014). In brief, mice were exposed to N-Methyl-N-nitrosourea (MNU, Sigma Chemicals), which was dissolved in distilled water at a concentration of 240 ppm and freshly prepared twice per week for administration in drinking water in light-shielded bottles ad libitum. MNU was administered in the drinking water starting at 4 weeks of age and continued from the next 10 weeks followed by euthanization at age 12 months. PEITC was administered through an AIN-76A diet (3–5 µmol PEITC/g diet) either during or following administration of MNU. Mice were euthanized at age of 12 months.

### Treatment of Phenethyl Isothiocyanate in Genetically Engineered Mouse Model of Gastric Cancer

The transgenic insulin-gastrin mice (INS-GAS mice) that over-express gastrin develop spontaneously gastric cancer were generated as previously described (Zhao et al., 2014). Mice received PEITC through an AIN-76A diet (3–5 µmol PEITC/g diet) for 10 weeks or standard pellet food (RM1801002, Scanbur BK AS). Mice were euthanized at the age of 12 months.

### Tissue Sampling

The stomachs were removed, opened along the greater curvature, washed in 0.9% (w/v) NaCl, and pinned flat on a petri-dish-silicone board. Each stomach was photographed digitally; the tumor profiles in both anterior and posterior sides of the stomach were drawn separately and subjected to morphometric analysis of the volume density (expressed as the percentage of glandular volume occupied by the tumor) using point-counting technique with a test grid comprised of a 1.0 cm square lattice. This grid was placed over each photograph (40 cm^2^ × 30 cm^2^), and the numbers of test points overlying the tumor and gastric glandular area were determined.

### Chemicals and Reagents

Phenethyl isothiocyanate (PEITC, Sigma Aldrich, United States, cat. no. 253731-5G), Benzyl isothiocyanate (BITC, Sigma Aldrich, Poland, cat. no. 252492-5G) and Allyl isothiocyanate (AITC, Sigma Aldrich, Germany, cat. no. 377430-100G) were dissolved in 100% dimethylsulfoxide (DMSO) to working concentrations. Cisplatin (Wako Pure Chemical Industries Ltd., Osaka, Japan, cat. no. 033-20091, Lot. SAQ1693 or TOCRIS Bioscience, Abingdon, United Kingdom, cat. no. 2251) was dissolved in PBS (Nacalai Tescue, Japan, cat. no. 14249-24) under gentle warming, and 5-fluorouracil (Sigma Aldrich, China) was dissolved in 100% DMSO. The following cell culture supplements were used: DMEM (Nacalai tesque, Japan, cat. no 08456-65); Fetal Bovine Serum (FBS; ThermoFisher Scientific, United States), antibiotic-antimycotic solution containing penicillin, streptomycin and amphotericin B (Nacalai tesque, Japan, cat. no. 02892-54), Penicillin-streptomycin solution (Sigma Aldrich, Oslo, Norway, cat. no. P4333-100ML), RPMI-1640 medium (Sigma Aldrich, Norway, cat. no. R8758-500ML with 0.3 g/L (2 mM) glutamine), DMEM (Gibco, ThermoFisher Scientific, Oslo, Norway, cat. no. A14430-01 without L-glutamine, d-glucose, phenol red and sodium pyruvate); dialyzed FBS (Life technologies, United States, Cat. no 26400-036); L-glutamine (Sigma Aldrich, Oslo, Norway, cat. no G7513); Sodium pyruvate (Sigma Aldrich, Oslo, Norway, cat. no. S8636). For cell cycle analysis: Propidium iodide (P1, Sigma Aldrich, Oslo, Norway, cat. no P4170-10MG); Triton-X (Sigma Aldrich, Oslo, Norway, cat. no. T9284), RNase A (Sigma Aldrich, Oslo, Norway, cat. no R4875-100MG). For GSH determination: 5-Sulfosalicylic acid (SSA) solution (5.0%); For Western Blot: RIPA cell lysis buffer (Pierce) containing 0.1% MG132 Proteasome Inhibitor (Cayman Chemical), 1.0% Protease inhibitor cocktail (Sigma Aldrich) and 10% PhosStop Phosphatase Inhibitor Cocktail (Roche). Antibodies: Primary antibody mouse monoclonal anti human p53 clone DO-1 (Santa Cruz: sc-126); Mouse monoclonal anti-β-actin clone; Anti-mouse IgG HRP-linked whole Ab sheep (GE Healthcare: NA931)/Anti-Rabbit IgG, HRP-linked whole Ab donkey (GE Healthcare: NA934).

### Cell Culture

Human gastric carcinoma cancer cell lines AGS and MKN45 were maintained in Dulbecco’s Modified Eagle’s Medium (DMEM; Nacalai tesque, Japan) supplemented with 10% fetal bovine serum and 1% antibiotic-antimycotic solution containing penicillin, streptomycin and amphotericin B in a humidified incubator holding 5% CO_2_ and 37°C. Human gastric carcinoma cancer cell lines MKN74 and KATO-III were maintained in RPMI-1640 medium supplemented with 10% FBS and 1% penicillin-streptomycin solution. Passages were performed when cultures reached 70–80% confluency. For the studies investigating glutamate and glutamine contents, DMEM containing 4.5 g/L glucose, 2 mM glutamine or 0.2 mM glutamine and 1 mM sodium pyruvate supplemented with dialyzed FBS was used.

### Proliferation Assay

For proliferation assay, 1,500 cells of AGS, 2,500 of MKN45 or MKN74 or 3,000 cells of KATO-III were seeded in 96-well plates before incubated overnight allowing cells to confluate. Treatments were always accompanied by vehicle controls (*n* = 12) on each plate (0.05% DMSO). Cells were treated with AITC (Sigma Aldrich, Germany), BITC (Sigma Aldrich, Poland), PEITC (Sigma Aldrich, United States), cisplatin (Wako Pure Chemical Industries Ltd., Japan) or (Tocris, Norway) and 5-fluorouracil (Sigma Aldrich, China) as indicated in the text. Following treatment, Cell Count Reagent SF (Nacalai tesque, Japan) was added according to providers’ instructions to each well before mixing and incubating for 1.0–1.5 h. Proliferation was determined by measuring absorbance at 450 nm using a well plate reader. Defined DMEM was used to perform experiments with controlled levels of glutamine and glucose.

### Cell Cycle Analysis

Human gastric cancer cells KATO-III were seeded as 2.5 × 10^5^ cells in 6-well plates and incubated over two nights before treated with 0, 5 or 10 µM PEITC for 12 and 24 h or PEITC (0, 5, 10 µM) together with cisplatin (25 or 50 µM) for 24 h. Cells were harvested, resuspended in PBS and fixated in chilled ethanol (−20°C, 70%, Kemetyl Norway) for minimum 15 min. Cells were then pelleted and resuspended in freshly prepared propidium iodide (PI) staining solution (0.25% Triton- X-100, 50 μg/ml PI and 200 μg/ml RNase A) for 30 min. Cell cycle distribution was analyzed using a FACS Canto flow cytometer counting 2 × 10^4^ cells per sample in triplicates. Cell cycle distribution was acquired from the obtained histograms using FACS Diva software.

### Morphology

To study the effect of PEITC on cells, AGS and MKN74 cells were seeded in T_25_ flasks (1.5 × 10^5^ cells per flask) and left for overnight incubation before treating with 5–20 µM PEITC or vehicle control (0.1% DMSO) for 24 h. The cultures were then observed and pictures captured through an inverted microscope in phase contrast mode.

### Total GSH Determination

Total cellular glutathione level was determined in PEITC, AITC or BSO-treated AGS cells. Cells were seeded in T_25_ flasks (1.5 × 10^5^ cells per flask) and incubated overnight prior to treatment. The cultures were treated with either 10–20 µM PEITC, 50–100 µM AITC, or 0–100 µM BSO or vehicle control (0.1% DMSO) for 3 or 6 h. The doses were based on IC_50_-range and previous literature. Each treatment was performed in quadruples. Cells were harvested and centrifuged (1,500 rpm, 5 min) before determination of total cellular glutathione using a commercial glutathione assay kit (Sigma, United States) according to manufacturers’ instructions. Briefly, cell pellets were deproteinized in 5-sulfosalicylic acid (SSA) solution (5%), vortexed and snap-freezed (3 times in total) before centrifugation (1,500 rpm, 5 min). Supernatants were transferred to clean tubes and stored on ice until analysis. 10 µl from each sample was applied to a 96—well plate in separate wells in duplicates and mixed together with 150 µl reaction mixture containing 95 mM potassium phosphate buffer (pH 7), 0.95 mM EDTA, 0.031 mg/ml DTNB, 0.115 units/ml glutathione reductase and 0.24% 5-sulfosalicylic acid. Finally, 50 µl of NADPH solution (0.16 mg/ml, resulting in final concentration of 0.038 mg/ml (48 µM) NADPH) was added to each well and mixed. Immediately after mixing, a kinetic read was performed in 1 min intervals for 5 min at 412 nm using a spectrophotometric plate reader in order to detect the formation of the yellow product 5-thio-2-nitrobenzoic acid (TNB).

### Glutamate/Glutamine Determination

For glutamate/glutamine detection, AGS cells were seeded in 24-well plates (1.0 × 10^4^ cells per well) and incubated over night to attain confluency. The cultures were then treated with PEITC (10–30 µM) and AITC (50–200 µM) for 2–24 h in defined DMEM containing dialyzed FBS before samples were collected and analyzed for glutamate and glutamine content. Determination of glutamate/glutamine was performed using a detection kit (Sigma, United States) following the manufacturers’ instructions. Briefly, from each sample to be analyzed, one sample was prepared for estimating endogenous glutamate, and one sample was prepared for estimating endogenous glutamate and glutamate converted from glutamine based on an initial deamination reaction catalyzed by glutaminase of the samples. All samples were then mixed with glutamic dehydrogenase which generates α-ketoglutarate and converts NAD^+^ to NADH which was detected spectrophotometrically at 340 nm. Glutamate content was then calculated using a standard curve, whereas glutamine content was calculated by subtracting the endogenous glutamate concentration from the total concentration of endogenous glutamate and glutamine-derived glutamate.

### Spheroid 3D Culture

AGS cells were seeded in 96-well plates (1,500 cells per well) with U-shaped bottoms with surface that prevents cells from attaching to the surface (Sumitomo Bakelite Co. Ltd., Japan). The cells were then incubated for 1 day to allow the cells to generate a spheroid-like structure before these spheroids were treated with PEITC (0–50 µM) for 48 h. After treatment, proliferation was assayed as described above.

### Western Blot

Western Blot from whole cell extract was performed in order to investigate the presence of protein p53. Cell extracts were prepared using ice-cold RIPA cell lysis buffer (Pierce) containing 0.1% MG132 Proteasome inhibitor (Cayman Chemical), 1% Protease inhibitor cocktail (Sigma Aldrich) and 10% PhosStop phosphatase inhibitor Cocktail (Roche). Bicinchoninate protein quantification (BCA) assay (Nacalai Tesque) was performed in order to determine protein concentrations in the cell lysates prior to SDS PAGE. Samples were denatured in sample buffer (4x) (NuPAGE LDS, Novex, Life Technologies, pH 8.4) with 5% 2-mercaptoethanol at 100°C for 10 min. Five microgram of protein or molecular weights marker were loaded into the lanes on the SDS PAGE gel and run in MOPS running buffer (NuPAGE, Life Technologies, pH 7.7) for 5 min at 150 V followed by 40 min at 200 V. After electrophoresis, gels were blotted onto polyvinylidene difluoride (PVDF) membranes in NuPAGE transfer buffer (Life Technologies). Block ACE solution (DS Pharma Biomedical) was used to block the membrane for 1 h at room temperature. Primary antibody mouse monoclonal anti human p53 clone DO-1 (1:200, Santacruz: sc-126) was added to the membrane and incubated overnight at 4°C. Mouse monoclonal anti-β-actin clone, which recognize β-actin, was used as internal standard. The membrane was washed in tris-buffered saline with 0.5% Tween 20 (TBST) followed by incubation with secondary antibody anti-mouse IgG HRP-linked whole Ab sheep (1:500) (GE Healthcare: NA931)/Anti-Rabbit IgG, HRP-linked whole Ab donkey (1:500) (GE Healthcare: NA934) for 1 h at room temperature. Finally, chemiluminescence capturing using Clarity Western ECL substrate (Bio-RAD) was applied, and images were acquired using a ImageQuant LAS 500 system (GE Healthcare). Quantification of p53 band area was performed in Image studio Lite (LI-COR Biosciences).

### Statistical Analysis

Values are expressed as means ± SEM in *in vivo* experiments. Pairwise comparisons between experimental groups were done using one-way ANOVA with Dunnett’s test (1-sided) or student’s t-test between INS-GAS mouse GC tumors with *vs*. without PEITC. In *in vitro* experiments, cell proliferation is represented by means of *n* = 3-6 replicates/treatment ± SD. IC_50_ values were calculated from sigmoidal regression curve fitting using variable slope on normalized response from log (10)-transformed x-values (GraphPad Prism v.6). Standard deviation (SD) values (%) were omitted from cultures with 98% or higher inhibited growth as these yielded non-representatively high SD values. Cell cycle distribution was analyzed using one-way ANOVA on normally distributed data with Dunnett’s 2-sided post hoc test *vs*. control groups. All tests were with a significance cutoff of *p* < 0.05.

## Results

**FIGURE 1:**
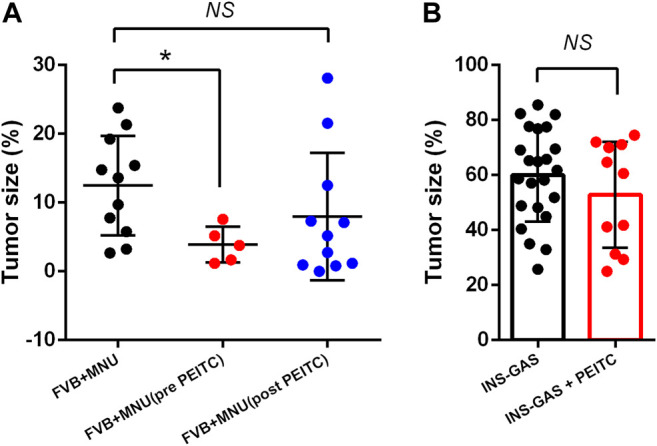
Tumor size of gastric cancer in mice that completed the study period of 45–50 weeks. FVB mice received MNU for 10 weeks (*n* = 11) or MNU together with PEITC (3–5 µmol PEITC/g diet) for 10 weeks (*n* = 5) or post MNU treatment (*n* = 11) **(A)** and in INS-GAS mice with or without PEITC (3–5 µmol PEITC/g diet) for 10 weeks **(B)**. Mean ± SEM. Tumor size expressed as volume density (% of glandular area occupied by tumor). ANOVA + Dunnett’s test (1-tailed) in **(A)**, student *t*-test in **(B)**. *p < 0.05; NS, not significant; PEITC, phenethyl isothiocyanate; MNU, N-methyl-N-nitrosourea.

Two mouse models of gastric cancer were used, i.e., MNU-induced gastric cancer (MNU mice) and genetically engineered spontaneously gastric cancer (INS-GAS mice). Body weight of mice with or without PEITC increased due to aging of the mice but was not affected by PEITC treatment during the period of experiment (10 weeks). Tumour size of gastric cancer was significantly reduced by PEITC when given during MNU but neither after MNU, nor in INS-GAS mice (Figures 1A,B).

**FIGURE 2:**
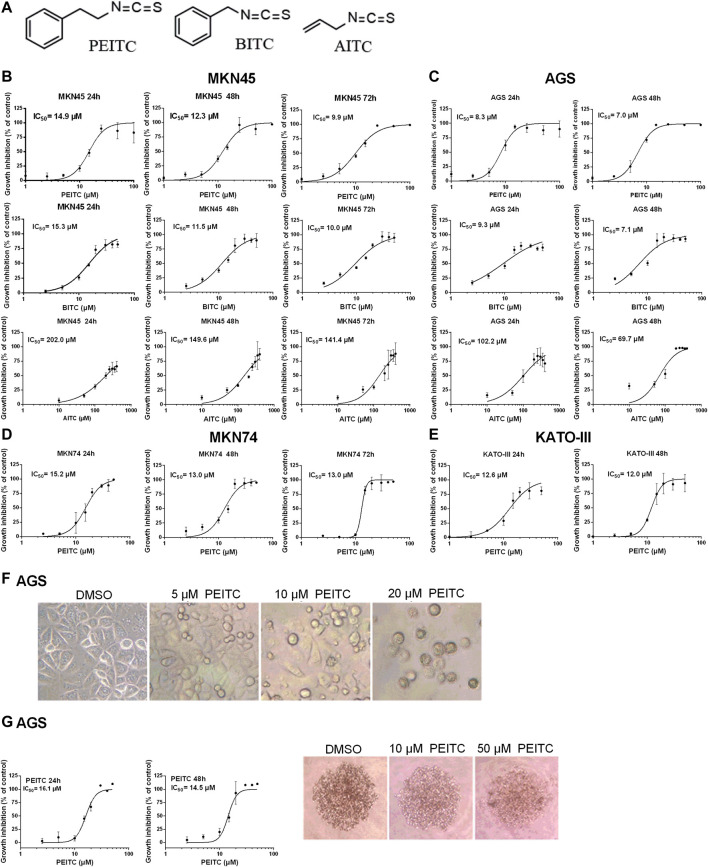
Chemical structures of ITCs **(A)** and proliferation dose-response curves of gastric cancer cell lines MKN45 **(B)** and AGS **(C)** when treated with PEITC (1–100 µM), BITC (2.5–50 µM) and AITC (10–400 µM) for 24, 48 and 72 h in medium containing 1.0 g/L (5.6 mM) glucose and 0.584 g/L (4 mM) glutamine. Values represent means of *n* = 3–6 replicates relative to vehicle control (0.1% DMSO), and IC_50_ values were calculated from the logistic sigmoidal regression curves shown. Standard deviation (SD) values were omitted from cultures with 98% or higher inhibited growth as these yielded non-representatively high SD values. Proliferation dose-response curves of gastric cancer cell lines MKN74 and KATO-III when treated with PEITC (1–50 µM) **(D, E)**. Morphology of AGS cells affected by ITC-treatments **(F)**. Proliferation and morphology of spheroid 3D cultures of AGS cells treated with PEITC for 24–48 h **(G)**. PEITC, phenethyl isothiocyanate; BITC, benzyl isothiocyanate; AITC, allyl isothiocyanate; DMSO, dimethylsulfoxide.

To demonstrate the cytotoxicity of ITCs in gastric cancer, four human gastric carcinoma cell lines were used; MKN45, AGS, MKN74 and KATO-III. Aromatic PEITC, BITC or aliphatic AITC resulted in a time and dose-dependent inhibition of cell proliferation (Figures 2A–E). The aromatic ITCs displayed a higher potential in inhibiting cell proliferation in both MKN45 and AGS compared to AITC. The MKN74, MKN45 and KATO-III cells proved to be more tolerant to ITC-treatment than the AGS cells in terms of IC_50_-values. All cell lines showed alterations in cell morphology by ITC-treatments with a gradual increase in non-confluent cells with increasing ITC-doses as demonstrated by PEITC-treated AGS cells (Figure 2F). A spheroid 3D culture of AGS cells treated with PEITC for 24 and 48 h showed decreased growth upon increasing doses (Figure 2G).

**FIGURE 3:**
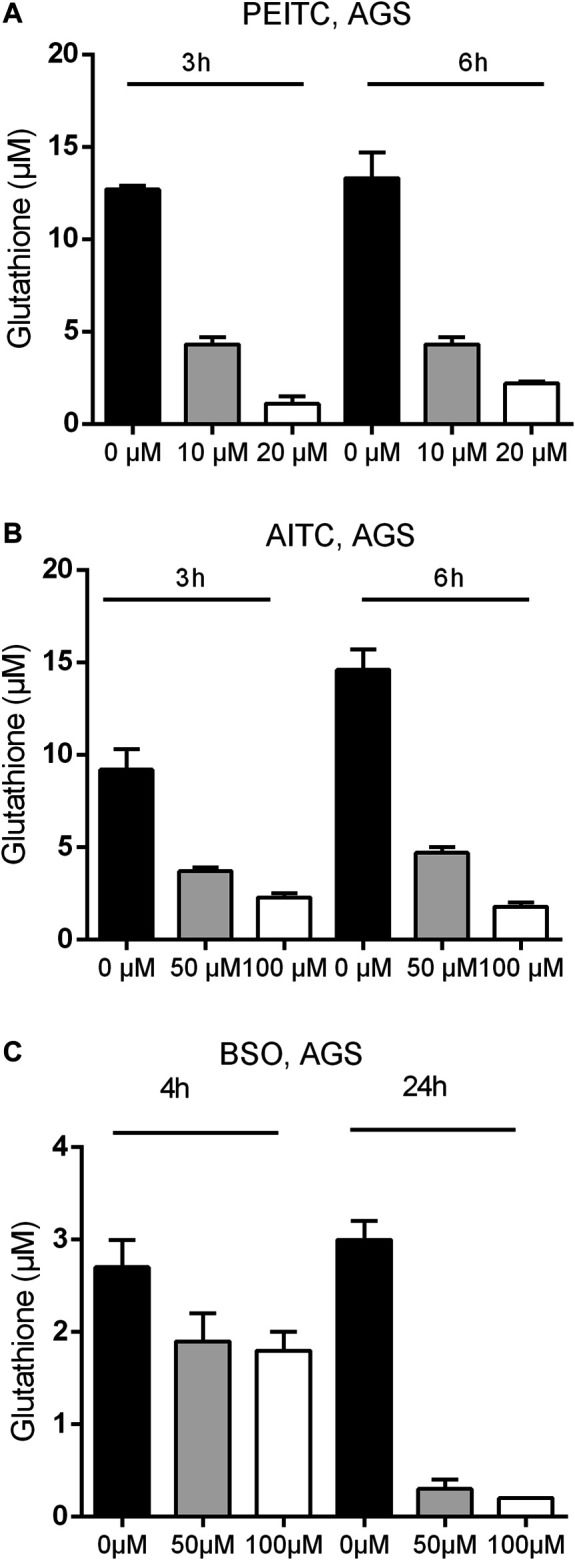
Glutathione concentration upon 3 and 6 h treatments with PEITC **(A)** or AITC **(B)** on AGS cells and upon 4 and 24 h of treatment with BSO **(C)** on AGS cells. Mean + SD of *n* = 4 replicates/treatment. PEITC, phenethyl isothiocyanate; AITC, allyl isothiocyanate; BSO, Buthionine sulfoximine.

Due to the electrophilic central C-atom in the reactive –N=C=S group, ITCs are able to antagonize multiple targets including glutathione. We therefore next examined the GSH concentration upon PEITC and AITC treatment. GSH depletion was both time- and dose-dependent in AGS cells (Figures 3A,B). Additionally, the synthetic amino acid Buthionine sulfoximine (BSO) depleted GSH in time- and dose-dependent manner (Figure 3C).

**FIGURE 4:**
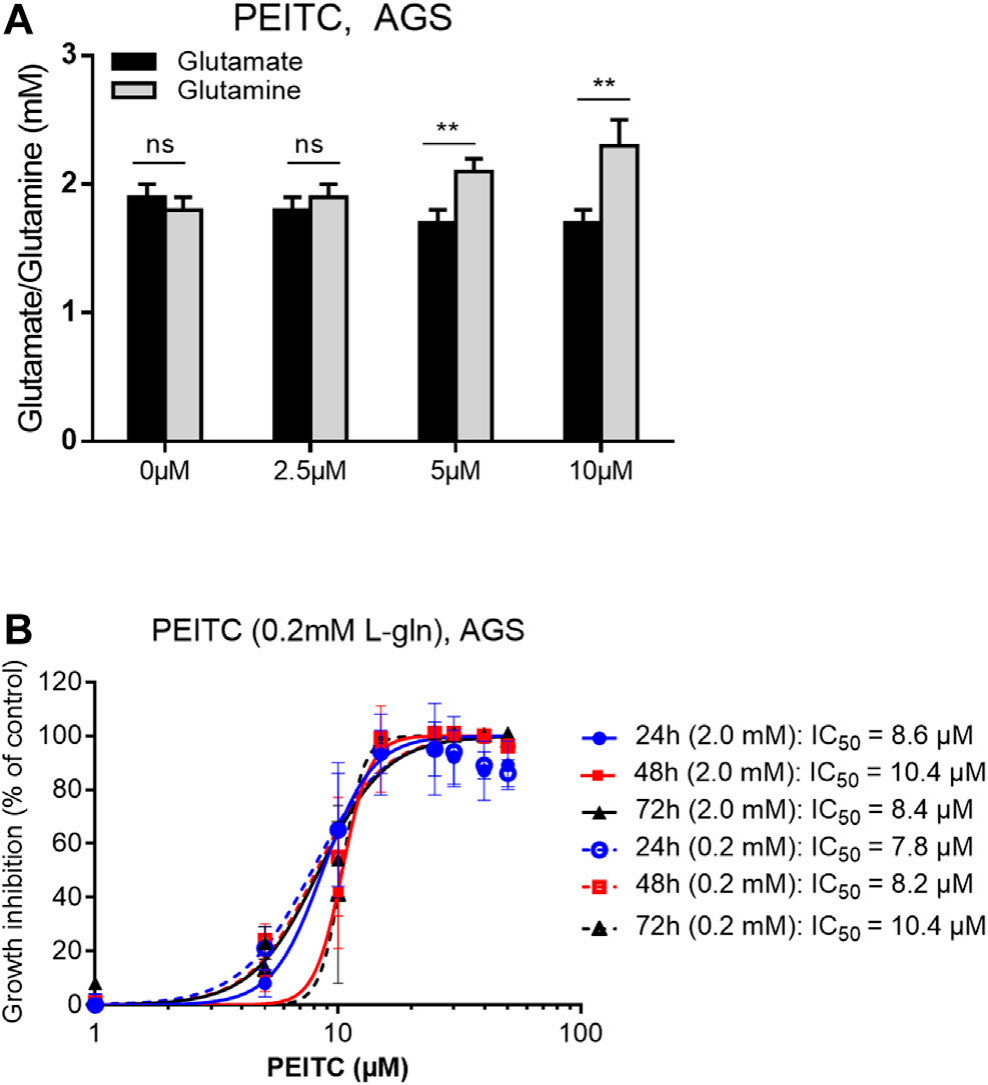
Glutamate/glutamine determination upon 6 h treatments with 0, 2.5, 5 or 10 µM PEITC on AGS cells **(A)**. PEITC treatment in L-glutamine-reduced (×10) DMEM medium **(B)**. Mean ± SD. Paired *t*-test in **(A)**. **p < 0.01; ns, not significant; PEITC, phenethyl isothiocyanate; L-gln, L-glutamine.

Reflected by the glutathione cycle, there are close relationships between glutathione and the levels of glutamine and glutamate in the cell-pool important for redox homeostasis. We next investigated the ratio between glutamine and glutamate after PEITC. PEITC increased the ratio of glutamine/glutamate in a dose-dependent manner, and furthermore inhibited cell proliferation in glutamine-reduced medium in a concentration-depended manner (Figures 4A,B).

**FIGURE 5:**
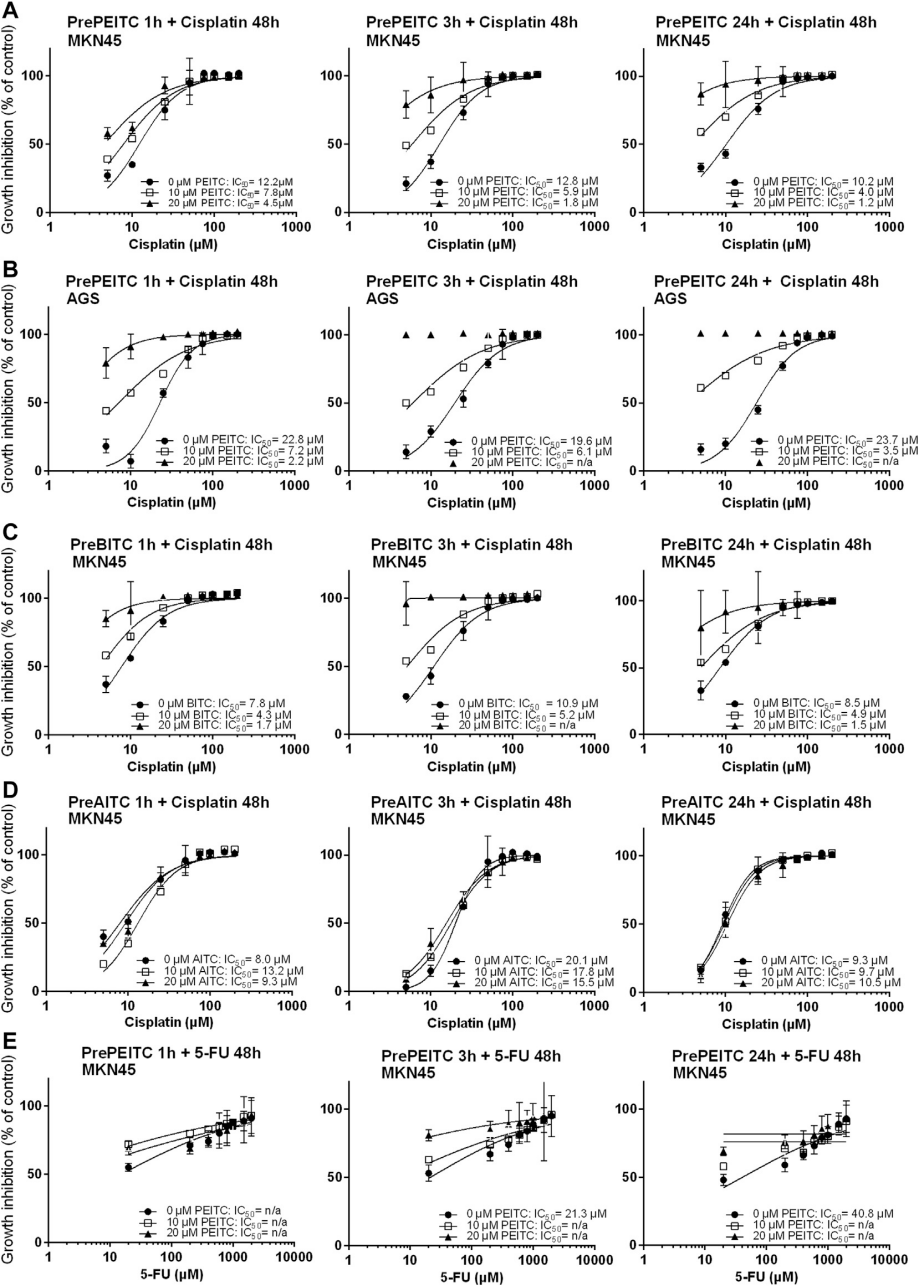
Inhibition of proliferation (relative to vehicle control, 3% PBS) in cell cultures of MKN45 pre-treated with 10 and 20 µM PEITC for 1, 3 and 24 h before treated with 5–200 µM Cisplatin for 48 h **(A–E)**. (**B, C)** same as **(A)** but with pre-treatment with BITC or AITC instead of PEITC, respectively. **(D)** same as in **(A)** using AGS cells instead of MKN45. **(E)** same as in **(A)** but treating cells with 5-fluorouracil for 48 h instead of cisplatin following pre-treatment with PEITC. Values represent mean of *n* = 3–6 replicates. SD values were omitted from cultures with 98% or higher inhibited growth as these yielded non-representatively high SD values. PEITC: phenethyl isothiocyanate; BITC, benzyl isothiocyanate; AITC, allyl isothiocyanate.

The GSH-pool is an important factor for the cancer cells to maintain redox homeostasis. By depleting cells of glutathione, we hypothesized that ITCs would enhance the *in vitro* cytotoxicity of cisplatin. To investigate the potential effects of ITCs, AGS and MKN45 cells were pretreated with PEITC, BITC or AITC for 1, 3 or 24 h followed by cisplatin or 5-FU treatment for 48 h (Figures 5A–E). Pretreatment with 20 µM PEITC in MKN45 cells for 1 h lowered the IC_50_ of cisplatin by 2.7-fold, while pretreatment for 3 h lowered the IC_50_ of cisplatin 7-fold. After 24 h, the reduction in IC_50_ was 8.5-fold (Figure 5A, third panel). Pre-treatment with 20 µM PEITC in AGS cells showed 10-fold reduction after 1 h (Figure 5B). A similar observation was made for the BITC compound, where 20 µM BITC showed 4.6 and 5.7-folds reductions in IC_50_ after 1 and 24 h, respectively (Figure 5C). The aliphatic AITC failed to induce the synergistic effects with cisplatin, only lowering the IC_50_ by 1.3-fold after 3 h or even showing increased IC_50_ upon pretreatment (1 and 24 h, Figure 5D). Substituting cisplatin by 5-FU did not achieve the same inhibition using PEITC (Figure 5E).

**FIGURE 6:**
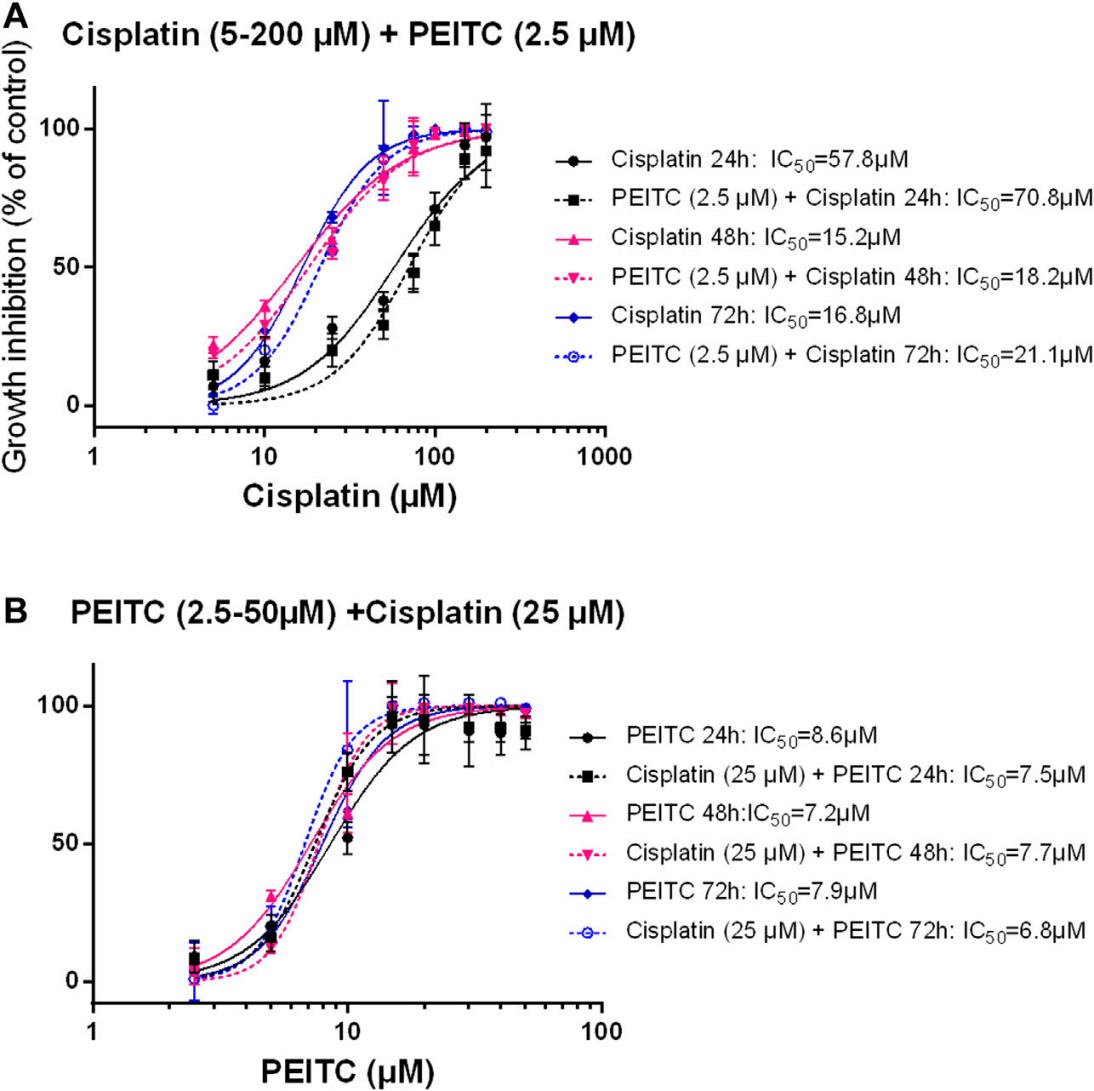
Simultaneous treatment with PEITC and Cisplatin as different concentrations of cisplatin **(A)** and different concentrations of PEITC **(B)**. Mean ± SD. PEITC, phenethyl isothiocyanate.

Simultaneous treatments with PEITC (2.5 µM) and cisplatin at increasing doses showed no additional inhibitory effect or even had antagonistic effect as reflected in increased IC_50_ values when PEITC was added (Figures 6A,B).

**FIGURE 7:**
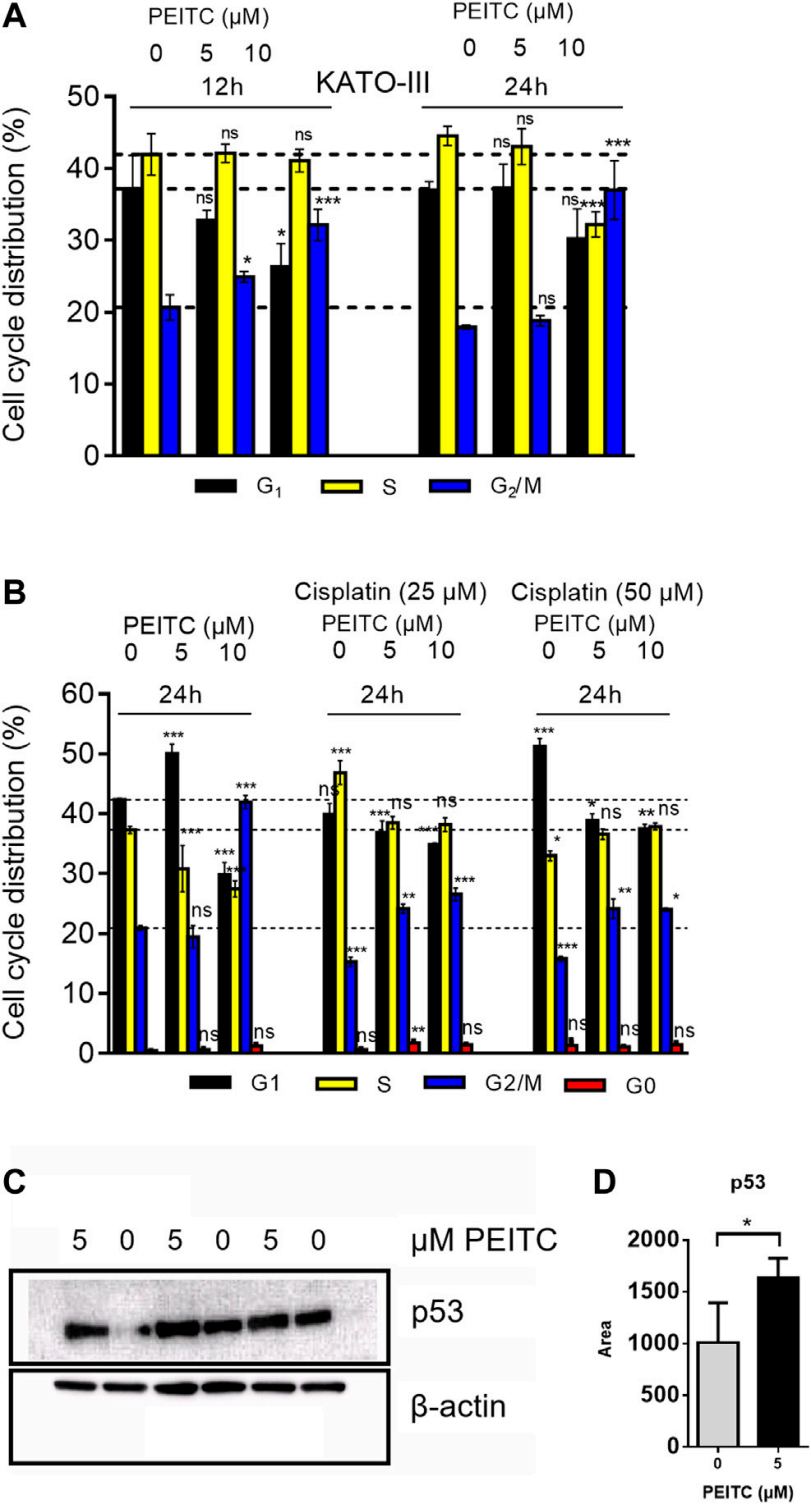
Cell cycle analysis of PEITC **(A)** and PEITC + cisplatin **(B)** in KATO-III cells. Analyzed using FACS Canto cell cycle sorter with 20,000 cell reads/sample. Distribution is derived from histograms in FACS Diva software. Mean ± SD of *n* = 3 replicates/treatment. ANOVA with Dunnett’s test (2-sided) vs. control groups was used. Western blot assessment of proteins p53 and β-actin (control) in response to PEITC were included in triplicates **(C)**. p53 band area were quantified in Image Studio Lite **(D)**. Bars represent means ± SD. Independent samples *t*-test (one-tailed) between 0 and 5 µM *: *p* < 0.05.

Cell cycle distribution of KATO-III cells upon 12 and 24 h treatments with PEITC resulted in G_2_/M phase arrest (Figure 7A). However, when treated with 0, 5 or 10 µM PEITC together with 0, 25 or 50 µM cisplatin, a decrease in G_1_ phase was accompanied by increase in G_2_/M phase and slight increase in apoptotic cells (reflected by sub G_1_/G_0_ phase increase) (Figure 7B). Treatment of AGS cells with 0 or 5 µM PEITC for 24 h showed increased level of protein p53 as determined by Western Blot (Figures 7C,D).

## Discussion

Long-term exposure to *H. pylori* is associated with progression of precancerous lesions in the stomach and infected individuals may benefit from successful *H. pylori* eradication, and population-based chemopreventive strategy of *H. pylori* eradication is still under the development (Tan and Wong, 2013; Mera et al., 2018). Other strategies using drugs, such as non-steroidal anti-inflammatory drugs and statins, have also been suggested (Ford, 2011). In the present study, we found the chemopreventive effects by PEITC in chemically induced (MNU) animal model of gastric cancer. Interestingly, the chemopreventive effects were neither seen when PEITC was given after the tumor initiation by MNU nor in genetically induced (INS-GAS) gastric cancer. Thus, it is unlikely that PEITC interacts directly with MNU on one hand, but on the other hand, PEITC may act on gastric epithelial cells to prevent the initiation of tumorigenesis as it has been suggested that PEITC induce apoptosis, inhibits cell cycle progression and inhibits angiogenesis (Mitsiogianni et al., 2019). It is known that the regulation of apoptosis by ITC is achieved primarily through mitochondrial cytochrome c release, regulation of the Bcl-2 family, MAPK signaling and subsequent activation of caspases, responsible for the initiation and execution of apoptosis. Specifically, AITC and phenyl-ITC (PITC) inhibit TNF (extrinsic apoptosis), generating a mycelial inhibition for several months, while BITC and PEITC induce a cytochrome c release-dependent type of apoptosis from mitochondria (intrinsic apoptosis) that generates a mycelial inhibition that lasts only for a few days. The differences in the fungistatic effect of ITC are possibly due to the type of apoptosis induced. It appears that significant portion of the chemopreventive effects of ITCs might be associated with the inhibition of the metabolic activation of carcinogens by cytochrome P450s (Phase I), coupled with strong induction of Phase II detoxifying and cellular defensive enzymes. Inductions of Phase II cellular enzymes are largely mediated by the antioxidant responsive element (ARE), which is regulated by the transcriptional factor (Nrf2). Additional potent regulatory mechanisms of Nrf2 include the different signaling kinase pathways (MAPK, PI3K, PKC and PERK) as well as other non-kinase dependent mechanisms. Moreover, apoptosis and cell cycle perturbations appear to be yet another potential chemopreventive mechanisms elicited by ITCs, especially with respect to the effects on pre-initiated or initiated tumor cells. Finally, modulation of other critical signaling mediators, including the NF-κB and AP-1 by a wide array of chemopreventive agents including ITCs might also contribute to the overall chemopreventive mechanisms (Keum et al., 2004).

Although surgery-related outcomes for treatment of gastric cancer, e.g., minimally invasive surgery techniques, continue to improve, the best regimen of either mono- or combination chemotherapy treatments still needs to be improved (Leiting and Grotz, 2019). In fact, the survival benefit of combinations of 5-fluorouracil (5-FU) with leucovorin, etoposide, methotrexate, doxorubicin, epidoxorubicin, cisplatin or oxaliplatin has been demonstrated (Sjoquist and Zalcberg, 2015). The results of the present study showed that there were time- and dose-dependent proliferative inhibitions by PEITC, BITC or AITC *in vitro* using the human cancer cell lines MKN45, AGS, MKN74 and KATO-III which were derived from intestinal and diffuse types of gastric carcinoma. Furthermore, the results of the present study showed that PEITC depleted intracellular levels of GSH and induced G_2_/M arrest. It is well established that ITCs conjugate with GSH which is a linear tripeptide of L-glutamine, L-cysteine, and glycine. GSH is the main antioxidant metabolite in the cell and provides electrons for enzymes such as glutathione peroxidase, which reduce H_2_O_2_ to H_2_O. GSH is crucial for cell proliferation, cell cycle progression and apoptosis and to protect cells from toxic insult by detoxifying toxic metabolites of drugs and ROS (Aquilano et al., 2014; Diaz-Vivancos et al., 2015). The results of the present study showed that intracellular GSH depletion upon PEITC and AITC treatment was both time-and dose-dependent, suggesting gastric cancer are susceptible to glutathione depletion. In fact, it was also reported that combined targeting of the epidermal growth factor receptor effector AKT and the glutathione antioxidant pathway mimicked Nrf2 ablation to potently inhibit pancreatic cancer, representing a promising synthetic lethal strategy for treating pancreatic cancer (Chio et al., 2016). This was in line with the results of the present study showing that the synergistic effect of PEITC took place when it was given prior to cisplatin but not simultaneously with cisplatin, as it needs to deplete the intracellular pool of glutathione in order to achieve cell cycle arrest in response to cisplatin. Of note, the results of the present study also showed that pretreatment with PEITC could enhance the cytotoxicity of cisplatin but not of 5-FU. This effect should be explained by the different mechanisms of action between cisplatin (forming DNA crosslinks) and 5-FU (inhibiting thymidylate synthase) (Larionova et al., 2019). It would be of interest to investigate further the effects of PEITC in combination with different chemotherapeutic agents (including cisplatin, 5-FU, paclitaxel, gemcitabine, and trabectedin) that have different mechanisms of action in order to explore the mechanism of PEITC and to find the best combination therapy.

Interference of ITC with microtubules have also been established as a contributor to cells stagnating in the G_2_/M-phase (Mi et al., 2009; Øverby et al., 2014). Buthionine sulfoximine (BSO), a synthetic amino acid, is an inhibitor of GSH synthesis on intracellular GSH levels (Griffith and Meister, 1979; Aldini et al., 2018). The results of the present study showed that BSO depleted GSH in a time- and dose-dependent manner and that PEITC-treatment altered the intracellular glutamine/glutamate ratio, providing a possible link between ITCs and amino acid metabolism. We suggested that the increase in glutamine but not glutamate levels shown in the present study could be attributed to compensatory mechanisms towards GSH replenishment in the cell when GSH level decreases. Indeed, a previous report has found that glutamine consumption correlated with glutathione excretion (Sappington et al., 2016).

It is known that elevated GSH levels are associated with tumor cell resistance to alkylating agents and platinum compounds (Estrela et al., 2006; Ortega et al., 2011; Bansal and Simon, 2018). Elevated GSH levels are observed in various types of tumors (Calvert et al., 1998). It has been suggested that high intracellular GSH level increases the antioxidant capacity and is thus conferring therapeutic resistance to cancer cells through the ability to resist oxidative stress which is a critical component of cisplatin cytotoxicity (Yu et al., 2018). We hypothesized that ITCs would enhance the cytotoxicity of cisplatin by depleting cells of glutathione, and indeed we found that PEITC and BITC but not AITC sensitized the gastric cancer cells to cisplatin. Conceivably, when the cell is depleted of GSH and oxidative stress is introduced using cytotoxic agents, a collapse in the antioxidant system eventually leads to cell death. Although reduction in GSH is proposed as a possible mechanism in the present study, it should be noticed that ITCs at sufficiently low doses might actually increase GSH levels as a consequence of ROS induction. Di Pasqua and colleagues described reduction of GSH as a less likely explanation to potentiating lung cancer cells by ITC but accredited the binding to tubulin as a more plausible explanation (Di Pasqua et al., 2010). In fact, PEITC and cisplatin have been co-administered using liposomal nanoparticles for treatment of non-small cell lung cancer (Sun et al., 2019). The efficacy potentiating of ITCs on existing chemotherapy has also been studied in cancers such as Barrett esophageal adenocarcinoma (Qazi et al., 2010), ovarian carcinoma (Stehlik et al., 2010), non-small cell lung carcinoma (Di Pasqua et al., 2010), prostate cancer (Xiao and Singh, 2010) and cervical cancer cells (Wang et al., 2011) in combination with drugs such as paclitaxel, MST-312, GRN163L, cisplatin and docetaxel. Thus, the results of the present study provide additional evidence in gastric cancer. The results of the present study also showed that PEITC induced cell cycle arrest in G_2_/M phase which was associated with increased p53 protein levels. p53 is one of the classical tumor suppressor genes that interferes with cell transformation events and plays a critical role in cell cycle control and induction of apoptosis (Ozaki and Nakagawara, 2011; Bykov et al., 2016; Bykov et al., 2018). It can be elevated in response to genotoxic agents, such as ionizing radiation, UV light, or chemicals. It has been shown that p53 elevation was required for PEITC-induced apoptosis (Huang et al., 1998).

However, some limitations of the present study should be noticed. First, we did not include additional animal groups, e.g., normal mice, MNU and INS-GAS mice that should be treated with PEITC or cisplatin alone and combination of PEITC plus cisplatin to explore the possibilities that PEITC may have differential effects on gastric cancer cells compared to normal gastric epithelial cells and that there is likely a synergistic anticancer effect *in vivo*. In fact, it has been showed that combining AITC with cisplatin reduced tumor volume in a mouse model of human lung cancer (Ling et al., 2015), thus this could also be a promising strategy in gastric cancer. Secondly, we did not investigate the molecular mechanism of action including signaling pathways of ITCs in combination with cisplatin, in gastric cancer cells. Third, we did not perform the combination of denervation and PEITC with or without chemotherapy, as initially planned. Forth, we did not further investigate the possible mechanism by which the only pretreatment with PEITC was effective against NMU-induced gastric cancer, and neither concomitant treatment nor administration of this agent after cancer development (either in NMU or INS-GAS mice) was successful. In addition to pre-initiated or initiated tumor cells as a possible target of PEITC (aforementioned), there are other possible hypotheses/explanations. It has been known that there are different windows for chemoprevention and therapeutic effects during the tumorigenesis from initiation, promotion and progression (Hanahan and Weinberg, 2011; Liu et al., 2015). It is also possible that the anti-cancer agents (e.g., ITCs) exhibit the effect on the initiation phase when given at a low dose and on the progression phase at a high dose. In the present study, PEITC (MW 163.24 g/mol) was given at 3–5 µmol/g diet in mice. Based on the pharmacokinetics of PEITC, the oral administration of PEITC at this dose level would reach a circulation level that is in a similar order of magnitude of IC_50_ (15 µM) *in vitro* but be a lower order of magnitude in gastric tissue (pmol/mg) (Reimer, 1972; Conaway et al., 1999). Fifth, it is still unclear why the synergistic effect was not obtained when PEITC and cisplatin were given simultaneously in the cell culture model. In fact, we failed to measure GSH levels because of heavily fluctuating potentiating effect. Fluctuating levels of GSH was found across our experiments measuring GSH concentration, where the intracellular GSH levels ranged between 3 and 10 µM GSH between experiments, adding to the complexity of GSH’s role in the observations. Finally, it should also be noticed that this study was carried out in the mouse models of gastric cancer and in the cell lines derived from human gastric cancer. It would be of interest to study the possible cytotoxic effects of ITCs in normal tissue and/or cell lines derived from normal healthy human stomach, e.g., cell line of HGaEpC, in the future. Taken together, it is still a challenge for future development of food products that contains high levels of edible ITCs for chemo-prevention and for being used during chemotherapy in patients with gastric cancer.

It would also be of interest to explore the possible efficacy’s potentiating role of ITCs on other therapies, such as targeted therapy and immunotherapy. In fact, combination of ERBB2 antagonist or RARA agonist was reported to be effective synergistic regimens for ERBB2 positive gastric cancer (Xiang et al., 2018). In clinical setting, the treatment options for advanced-stage gastric cancer are limited, despite an approval of two targeted agents, trastuzumab and ramucirumab. Consequently, the overall clinical outcomes for patients with advanced-stage gastric cancer remain poor. Numerous agents that are active against novel targets have been evaluated in the course of randomized trials; however, most have produced disappointing results because of the heterogeneity of gastric cancer (Kumar et al., 2018). Immunotherapy, e.g., immune checkpoint inhibitors (ICIs), has been tested in gastric cancer. Despite having good efficacy and safety profile, ICIs are clinically active only in small subset of patients and therefore, there is a huge unmet need to enhance their efficacy. Indeed, there are several ongoing clinical trials that are exploring the role of ICIs in various gastrointestinal cancers either as single agent or in combination with chemotherapy, radiation therapy, targeted agents or other immunotherapeutic agents, but not yet ITC (Mazloom et al., 2020).

## Conclusion

PEITC displayed anti-cancer effects, particularly when given before the tumor initiation, suggesting a chemopreventive effect in gastric cancer, and that aromatic ITCs potentiated the anti-cancer effects of cisplatin, particularly when given before cisplatin, suggesting a possible combination strategy in treatment of gastric cancer.

## Data Availability

The raw data supporting the conclusions of this article will be made available by the authors, without undue reservation.
